# Epidemiological Profile of the Pathologies of the Oral Cavity in a Peruvian Population: A 9-Year Retrospective Study of 18,639 Patients

**DOI:** 10.1155/2019/2357013

**Published:** 2019-02-03

**Authors:** Ángelo Sabogal, Jhonn Asencios, Ada Robles, Eloy Gamboa, José Rosas, Jorge Ríos, Frank Mayta-Tovalino

**Affiliations:** ^1^School of Stomatology, Universidad Privada San Juan Bautista, Lima, Peru; ^2^Professor of the Stomatology School, Universidad Privada San Juan Bautista, Lima, Peru; ^3^Research Professor and Academic Coordinator of the Master of Public Health Degree, Universidad Privada San Juan Bautista (Head: Prof. Dr. Frank Mayta-Tovalino), Lima, Peru

## Abstract

**Aim:**

To determine the epidemiological profile of oral diseases in a marginal urban Peruvian population.

**Methods:**

A 9-year retrospective study was conducted, analysing 18,639 clinical records from the María Auxiliadora Hospital between 2006 and 2015 with diagnoses of oral lesions using ICD-10 criteria. Clinical records were analysed for sex, tumour, periapical abscess and sinus, cysts of the oral region, other lesions of the oral mucosa and cavity, gingivitis and periodontal disease, dentofacial anomalies, diseases of pulp, etc.

**Results:**

Of 18,639 cases, the prevalence was higher in women for the following pathologies: unspecified dental caries (30.6%); impacted tooth in the category of disorders of tooth development and eruption (2.0%); pulpitis (6.8%) in the category of diseases of pulp and periapical tissues; temporomandibular joint (TMJ) disorders (1.5%) in the category of dentofacial anomalies; acute gingivitis (7.5%); radicular cyst (0.3%) in the category of cysts of the oral region; and periapical abscess without sinus (2.0%).

**Conclusions:**

We found a significant association between sex and different types of dental caries, disturbances in tooth eruption, diseases of pulp and periapical tissues, and dentofacial anomalies. The study also shows a statistically significant association between sex and gingivitis, periodontal disease, and periodontal abscess and sinus.

## 1. Introduction

The jaws and maxillofacial region are affected by pathologies including lesions that vary in location, histopathogenesis, and aetiology involving bone and soft tissues, with manifestations requiring additional clinical examinations and where pharmacological treatments prevent or at least minimise extensive surgical procedures with mutilations [1]. Normal dentition develops from the dental lamina, which is sensitive to disturbances, and the enamel does not regenerate after injury. Multiple factors affect the development of teeth, resulting in different lesions with hypoplasia, dysplasia, hypomineralisation, etc. [2]. For instance, some lesions derive from the enamel organ, dental lamina or epithelial rests of Malassez, and remnants from odontogenic epithelium affecting teeth and periodontal tissues [3–5]. These lesions include dental caries, cysts, tumours, and infections, which are the diseases with the highest prevalence and the main reason for consultation in dentistry in the population. They are divided into lesions of odontogenic and nonodontogenic origin; these anomalies are genetically inherited while others are acquired such as tumours and cysts [6–8].

On the other hand, some lesions arise in the jaws, are not tooth-related, and have no aetiological or histopathogenetic relationship with the ectoderm, facial tissues, etc. These lesions appear as an inflammatory reaction or lesions of unknown aetiology, where lesions of the oral mucosa and cavity prevail; tumours and infections are also part of this group according to another study [9].

There are few studies that describe the most prevalent oral diseases in a Peruvian population, which has great genetic biodiversity in Latin America. Therefore, the objective of this research is important because this 9-year retrospective study examined the prevalence of oral lesions in a marginal urban Peruvian population.

## 2. Participants and Methods

### 2.1. Participants of the Study

A cross-sectional, retrospective, and observational study was conducted during the years 2006 to 2015. We identified 18,639 electronic clinical records which were evaluated from the Department of Dentistry of the María Auxiliadora Hospital in Lima (Peru), registering the patients examined and diagnosed with different types of oral lesions, to know what the regional prevalence was of these pathologies in the Peruvian inhabitants of scarce economic resources.

### 2.2. Procedure

Oral lesions were identified using ICD-10 criteria (International Statistical Classification of Diseases and Related Health Problems). Variables such as sex, caries, disorders of tooth development and eruption, other diseases of hard tissues of teeth, and diseases of pulp and periapical tissues were collected for oral lesions of hard tissues. Similarly, variables such as gingivitis, and periodontal disease, other lesions of the oral mucosa and cavity, cysts of the oral region, periodontal abscess and sinus, and tumours were taken for odontogenic and nonodontogenic lesions of soft tissues.

The procedure for this research followed Strengthening the Reporting of Observational studies in Epidemiology (STROBE) guidelines. In addition, it was authorised by the Ethics Committee of Universidad Privada San Juan Bautista (approval code CEPB-FCS 0006).

### 2.3. Statistical Analysis

For the statistical analysis, we used frequency measurements and the chi-square test, establishing a level of significance of p <0.05. All statistical tests were performed with Stata software (version 12.0, USA)

## 3. Results

### 3.1. Study Population

Out of 18,639 electronic clinical records over 9 years, when evaluating the prevalence of oral lesions of hard tissues for the category of caries, 7,045 cases of unspecified dental caries (37.7%) were observed, followed by 1,221 cases of dentine caries (6.5%), being more common in females (30.6% and 3.8%, respectively). In addition, 125 cases of caries limited to enamel were found, among other pathologies, with a prevalence of <1% of the population. Regarding disorders of tooth development and eruption, there were 661 cases of impacted teeth (3.5%), being more common in females (2.0%); there were also 175 cases with disturbances in tooth eruption, 107 cases with disturbances in tooth formation, and other pathologies with a prevalence of <1% of the population. Regarding diseases of pulp and periapical tissues, there were 1,919 cases of pulpitis (10.2%), followed by 1,440 cases of retained dental root (7.7%) and 512 cases of pulp necrosis (2.7%), being more common in females (6.8%, 5.2%, and 1.4%, respectively), as well as other pathologies with a prevalence of <1% of the population. In relation to dentofacial anomalies, there were 341 cases of TMJ disorders (1.8%), being more common in females (1.5%); additionally, there were 113 cases of malocclusion, 47 cases of tooth position anomalies, and other pathologies with a prevalence of <1% of the population. There was a statistically significant association between sex and these evaluated categories (p <0.05). In the category of other diseases of hard tissues of teeth, there were 46 cases of other jaw diseases, 24 cases of deposits (accretions) on teeth, and other pathologies with a prevalence of <1% of the population. However, there was no statistically significant association between sex and this evaluated category (p >0.05) (Tables 1 and 2).

### 3.2. Prevalence

When evaluating the prevalence of different oral pathologies of soft tissues, in the category of gingivitis and periodontal disease, there were 1,648 cases of acute gingivitis (8.8%), followed by 677 cases of chronic periodontitis (3.6%) and 417 cases of chronic gingivitis (2.2%), being more common in females (7.5%, 2.3%, and 1.6%, respectively), as well as other pathologies with a prevalence of <1% of the population. Regarding cysts of the oral region, there were 93 cases of radicular cyst, 37 cases of developmental odontogenic cysts, and other pathologies with a prevalence <1% of the population, being more common in females. With regard to periodontal abscess and sinus, there were 712 cases of periapical abscess without sinus (3.8%), being more common in females (2.0%), followed by 153 cases of cellulitis and mouth abscess and other pathologies with a prevalence of <1% of the population. There was a statistically significant association between these evaluated categories and sex (p <0.05). In the category of other lesions of oral mucosa and cavity, there were 34 cases of other and unspecified lesions of the oral mucosa, in addition to other pathologies with a prevalence of <1% of the population. Similarly, in the category of tumours, there were only 3 cases of central giant cell granuloma, with a prevalence of <1% of the population. However, these two evaluated categories showed no statistically significant association with sex, p >0.05 (Tables 3 and 4).

## 4. Discussion

Oral pathologies are reasons for consultation, and according to the WHO, biofilm is an ecosystem that causes odontogenic infections. Different authors consider these lesions the major and most common oral pathologies in all age groups, being a reason for professional intervention [6–8]. The available literature indicates that the misdiagnosis of these lesions results in various diseases such as periodontitis, osteitis, and other lesions; also, nonodontogenic lesions cause diseases such as mucosal and gland infections and tumours [6, 10, 11].

Out of a total of 18,639 electronic clinical records from a Peruvian hospital, this study showed 8,404 cases of caries (45.1%), of which 1,221 cases (37.7%) corresponded to unspecified dental caries, being more common in females (30.6%), which is in agreement with the empirical evidence from studies in Germany and the USA, where there was a greater prevalence in females [12]. In another study [13], the prevalence of caries was 93.19% greater in females, with a sex ratio of 0.77. Previously, a high prevalence of caries (69%) was observed in university students in Hong Kong using the DMFT index, where female prevalence was higher than that of males like other studies [14, 15].

Regarding disorders of tooth development and eruption, there were 661 (3.5%) cases of an impacted tooth, being more common in women (n=391; 2.0%), with a male-to-female ratio of 1:1.3. This is similar to the results of a study in Turkey, where there were 1,117 cases (9.2%) with one or more impacted teeth and a male-to-female ratio of 1:1.4. However, these results contradict what was found in Tanzania, where a male-to-female ratio of 1.2:1 was recorded [16].

With respect to diseases of pulp and periapical tissues, the most common lesion was pulpitis (10.2%), followed by retained dental root (7.7%), pulp necrosis (2.7%), and other pathologies, such as acute and chronic apical periodontitis, with a prevalence of <1% of the population, being more common in females for all conditions. This differs from a study of 4,209 emergency patients in a university hospital, where 2,058 suffered odontogenic infections: most (45.0%) had apical periodontitis, 20.8% had abscesses, 17.3% had marginal periodontitis, 16.3% had pulpitis, and 5.8% had pericoronitis, with men being more affected than women [17].

In relation to dentofacial anomalies, the most prevalent condition was a TMJ disorder (1.8%), being more common in females (1.5%), which was consistent with findings from Finland where 18.5% of 1,962 patients presented with a TMJ disorder, being more common in females [18]. Also, within this category, there was a higher prevalence of females with tooth position anomalies and abnormalities of the size and form of teeth, which is in agreement with a previous study where 1,172 radiographs of 581 men and 586 women were reviewed, finding abnormalities of form, position, and number with 213 teeth anomalies, being more common in women [8].

In the category of gingivitis and periodontal disease, there were 1,648 cases of acute gingivitis (8.8%), followed by chronic periodontitis (3.6%) and chronic gingivitis (2.2%), being more common in females, unlike what was found in Jordan, where 76% were cases of gingivitis, 5.5% were chronic periodontitis, and 2.2% were severe periodontitis, the latter being more common in men than in women, with a male-to-female ratio [19] of 1.6:1. Our results are also contrast with previous studies where there was a higher prevalence of gingivitis in men [20, 21]. They also contradict the higher prevalence of periodontitis found in the adult male population in the USA [22].

Regarding cysts of the oral region, there were 93 cases of radicular cyst, 37 cases of developmental odontogenic cysts, and other pathologies with a prevalence of <1% of the population, being more common in females. These results differ from a study on the prevalence of odontogenic cysts in Sicily, where the most common lesions were radicular cysts (84.5%), followed by dentigerous odontogenic cysts (11.4%), with radicular inflammatory cysts being more common in males [23].

There are limitations such as a lack of literature related to the subject to verify results in some categories (diseases of hard tissues of teeth and other lesions of oral mucosa). The study results provide data and establish the prevalence of a disease and the most affected population, resulting in the predominance of females for almost all conditions. This study is important for its contribution to the institution for monitoring and reporting, aiming to conduct prevention campaigns and thus compare the results with future studies.

## 5. Conclusions

In conclusion, according to this 9-year retrospective study in a Peruvian hospital, females predominated in almost all pathologies, and a statistically significant association was found between sex and types of caries, disorders of tooth development and eruption, diseases of pulp and periapical tissues, and dentofacial anomalies.

A statistically significant association was also found between sex and gingivitis and periodontal disease as well as periodontal abscess and sinus. This study is pioneering because it determined the most prevalent oral pathologies at one of the main national hospitals in Peru, and its results are useful for oral and maxillofacial surgeons and pathologists.

## Figures and Tables

**Table 1:** Classification and distribution of oral lesions (hard tissues).

	**Diagnosis**	**Abbrev.**	**No.**	%
**Caries**	Not present	NP	10235	54.9
	Dentine caries	CD	1221	6.5
	Caries limited to enamel	CLE	125	0.6
	Dental caries, unspecified	DCU	7045	37.7
	Arrested dental caries	ADC	8	0.0
	Other dental caries	ODC	3	0.0
	Caries in pits and fissures	CPF	2	0.0

**Disorders of tooth development and eruption**	Not present	NP	17444	93.5
	Embedded tooth	EmbT	82	0.4
	Impacted tooth	ImpT	661	3.5
	Teething syndrome	TS	51	0.2
	Disorders of tooth development, unspecified	DTDU	17	0.0
	Hereditary disturbances in tooth structure, not elsewhere classified	HDTSNEC	9	0.0
	Disturbances in tooth eruption	DTE	175	0.9
	Supernumerary tooth	SpnT	77	0.4
	Disturbances in tooth formation	DTF	107	0.5
	Staining of teeth	ST	12	0.0
	Atrophy of edentulous alveolar ridge	AEAR	4	0.0

**Other diseases of hard tissues of teeth**	Not present	NP	18476	99.1
	Excessive attrition of teeth	EAT	10	00
	Hypercementosis	Hpcmt	12	00
	Deposits (accretions) on teeth	DAT	24	0.1
	Certain disorders of gingiva and edentulous alveolar ridge	CDGEAR	18	0.0
	Other diseases of jaws	ODJ	46	0.2
	Pathological resorption of teeth	PRT	5	0.0
	Complete tooth loss	CLT	15	0.0
	Congenital absence of teeth	CAT	8	0.0
	Exfoliation of teeth due to systemic causes	ETDSC	6	0.0
	Other specified disorders of teeth and supporting structures	OSDTSS	19	0.1

**Diseases of pulp and periapical tissues**	Not present	NP	14631	78.4
	Pulpitis	Plpts	1919	10.2
	Necrosis of pulp	NOP	512	2.7
	Pulp degeneration	PD	17	0.0
	Acute apical periodontitis of pulpal origin	AAPPlO	32	0.1
	Abnormal hard tissue formation in pulp	AHTFP	21	0.0
	Chronic apical periodontitis	CAP	29	0.1
	Retained dental root	RDR	1440	7.7
	Other and unspecified diseases of pulp and periapical tissues	OUDPPT	38	0.2

**Dentofacial anomalies**	Not present	NP	18077	96.9
	Malocclusion	Mlcs	113	0.0
	TMJ disorders	TMJD	341	1.8
	Anomalies of tooth position	ATP	47	0.2
	Anomalies of jaw-cranial base relationship	AJCBR	15	0.0
	Major anomalies of jaw size	MAJS	11	0.0
	Abnormalities of size and form of teeth	ASFT	10	0.0
	Dentofacial functional abnormalities	DfclFA	4	0.0
	Anomalies of dental arch relationship	ADAR	14	0.0
	Developmental disturbances of jaws	DDJ	1	0.0

**Table 2:** Nine-year retrospective study of the prevalence of hard tissue lesions.

			**Sex**			**Total**		***p*** **∗**
		**Female**		**Male**				
		N	%	n	%	N	%	
**Caries**	NP	6861	36.8	3374	17.9	10235	54.9	0.000
	CD	722	3.8	499	2.6	1221	6.5	
	CLE	70	0.3	55	0.2	125	0.6	
	DCU	5711	30.6	1334	7.1	7045	37.7	
	ADC	6	0.0	2	0.0	8	0.0	
	ODC	2	0.0	0	0.0	2	0.0	
	CPF	1	0.0	1	0.0	2	0.0	

**Disorders of tooth development and eruption**	NP	12708	68.1	4736	25.4	17444	93.5	0.000
	EmbT	55	0.2	27	0.1	82	0.4	
	ImpT	391	2.0	270	1.4	661	3.5	
	TS	29	0.1	22	0.1	51	0.2	
	DTDU	8	0.0	9	0.0	17	0.0	
	HDTSNEC	5	0.0	4	0.0	9	0.0	
	DTE	88	0.4	87	0.4	175	0.9	
	SpnT	25	0.1	52	0.2	77	0.4	
	DTF	55	0.2	52	0.2	107	0.5	
	ST	8	0.0	4	0.0	12	0.0	
	AEAR	2	0.0	2	0.0	4	0.0	

**Other diseases of hard tissues of teeth**	NP	13262	71.1	5214	27.9	18476	99.1	0.159
	EAT	8	0.0	2	0.0	10	00	
	Hpcmt	11	0.0	1	0.0	12	00	
	DAT	11	0.0	13	0.0	24	0.1	
	CDGEAR	12	0.0	6	0.0	18	0.0	
	ODJ	31	0.1	15	0.0	46	0.2	
	PRT	4	0.0	1	0.0	5	0.0	
	CLT	10	0.0	5	0.0	15	0.0	
	CAT	7	0.0	1	0.0	8	0.0	
	ETDSC	6	0.0	0	0.0	6	0.0	
	OSDTSS	13	00	6	0.0	19	0.1	

**Diseases of pulp and periapical tissues**	NP	59	57.7	3872	20.7	14631	78.4	0.000
	Plpts	1269	6.8	650	3.4	1919	10.2	
	NOP	279	1.4	242	1.2	512	2.7	
	PD	15	0.0	2	0.0	17	0.0	
	AAPPlO	22	0.1	10	0.0	32	0.1	
	AHTFP	15	0.0	6	0.0	21	0.0	
	CAP	19	0.1	10	0.0	29	0.1	
	RDR	981	5.2	459	2.4	1440	7.7	
	OUDPPT	24	0.1	14	0.0	38	0.2	

**Dentofacial anomalies**	NP	12957	69.5	512	2.7	18077	96.9	0.000
	Mlcs	67	0.3	46	0.2	113	0.0	
	TMJD	284	1.5	57	0.3	341	1.8	
	ATP	28	0.1	19	0.1	47	0.2	
	AJCBR	8	0.0	7	0.0	15	0.0	
	MAJS	8	0.0	3	0.0	11	0.0	
	ASFT	7	0.0	3	0.0	10	0.0	
	DfclFA	2	0.0	2	0.0	4	0.0	
	ADAR	10	0.0	4	0.0	14	0.0	
	DDJ	0	0.0	1	0.0	1	0.0	
	DfclAU	3	0.0	3	0.0	6	00	

*∗*Pearson chi-square test; significance level p <0.05.

**Table 3:** Classification and distribution of oral pathologies (soft tissues).

	**Diagnosis**	**Abbrev.**	**No.**	%
**Gingivitis and periodontal disease**	Not present	NP	15549	83.4
	Acute gingivitis	AG	1648	8.8
	Chronic gingivitis	CG	417	2.2
	Acute periodontitis	AP	151	0.8
	Chronic periodontitis	CP	677	3.6
	Gingival enlargement	GE	73	0.3
	Periodontal disease, unspecified	PDU	6	0.0
	Stomatitis	Stoma	31	0.1
	Mucocele of salivary gland	MSG	29	0.1
	Alveolitis	Alvlts	52	0.2
	Periodontitis	Perio	6	0.0

**Other lesions of oral mucosa and cavity**	Not present	NP	18564	99.5
	Leukoplakia and other disturbances of oral epithelium, including tongue	LODOEIT	9	0.0
	Other and unspecified lesions of oral mucosa	OULOM	34	0.1
	Sialadenitis	Sialo	4	0.0
	Oral submucous fibrosis	OSF	9	0.0
	Other diseases of lip and oral mucosa	ODLOM	4	0.0
	Irritative hyperplasia of oral mucosa	IHOM	2	0.0
	Hairy leukoplakia	HL	2	0.0
	Gingival and edentulous alveolar ridge lesions associated with trauma	GEARLAT	4	00
	Granuloma and granuloma-like lesions of oral mucosa	GGLLOM	3	00
	Glossitis	Glos	4	0.0

**Cysts of the oral region **	Not present	NP	18491	99.2
	Radicular cyst	RC	93	0.4
	Developmental odontogenic cysts	DOC	37	0.1
	Other cysts of the oral region, not elsewhere classified	OCORNEC	3	0.0
	Other cysts of the jaw	OCJ	15	0.0

**Periapical abscess and sinus**	Not present	NP	17694	94.9
	Periapical abscess with sinus	PAWS	77	0.41
	Periapical abscess without sinus	PAWtS	712	3.8
	Cellulitis and abscess of mouth	CAM	153	0.8
	Cellulitis of face	CF	1	0.0
	Abscess of salivary gland	ASG	2	0.0

**Tumour**	Not present	NP	18636	99.9
	Giant cell granuloma, central	GCGC	3	0.0

**Table 4:** Nine-year retrospective study of the prevalence of oral pathologies (soft tissues).

		**Female**	%	**Male**	%	**Total**	%	***p*** **∗**
		**N**		**n**		**N**		
**Gingivitis and periodontal disease **	NP	11008	59.0	4541	24.3	15549	83.4	0.000
	AG	1401	7.5	247	1.3	1648	8.8	
	CG	304	1.6	113	0.6	417	2.2	
	AP	89	0.4	62	0.3	151	0.8	
	CP	438	2.3	239	1.2	677	3.6	
	GE	50	0.2	23	0.1	73	0.3	
	PDU	3	0.0	3	0.0	6	0.0	
	Stoma	19	0.1	12	0.0	31	0.1	
	MSG	12	0.0	17	0.0	29	0.1	
	Alvlts	46	0.2	6	0.0	52	0.2	
	Perio	3	0.0	3	0.0	6	0.0	

**Other lesions of oral mucosa and cavity**	NP	13320	71.5	5235	28.0	18564	99.5	0.313
	LODOEIT	6	0.0	3	0.0	9	0.0	
	OULOM	18	0.0	16	0.0	34	0.1	
	Sialo	3	0.0	1	0.2	4	0.0	
	OSF	5	0.0	4	0.2	9	0.0	
	ODLOM	4	0.0	0	0.0	4	0.0	
	IHOM	1	0.0	1	0.0	2	0.0	
	HL	1	0.0	1	0.0	2	0.0	
	GEARLAT	2	0.0	2	0.0	4	00	
	GGLLOM	2	0.0	1	0.0	3	00	
	Glos	2	0.0	2	0.0	4	0.0	

**Cysts of the oral region **	NP	13284	71.5	5207	27.9	18491	99.2	0.046
	RC	58	0.3	35	0.0	93	0.4	
	DOC	21	0.1	16	0.0	37	0.1	
	OCORNEC	2	0.0	1	0.0	3	0.0	
	OCJ	9	0.0	6	0.0	15	0.0	

**Periapical abscess and sinus**	NP	12855	68.9	4839	25.9	17694	94.9	0.000
	PAWS	31	0.1	46	0.2	77	0.41	
	PAWtS	389	2.0	323	1.7	712	3.8	
	CAM	97	0.5	56	0.3	153	0.8	
	CF	1	0.0	0	0.0	1	0.0	
	ASG	1	0.0	1	0.0	2	0.0	

**Tumour**	NP	13372	71.1	5264	28.2	18636	99.9	0.494
	GCGC	1	0.0	2	0.0	3	0.0	

*∗*Pearson chi-square test; significance level p <0.05.

## Data Availability

The data used in the statistical analysis of this study will be available upon authorization of the corresponding managers of the university.
